# Association Between Online Self-Directed Learning Ability and Negative Emotions Among College Students During the COVID-19 Pandemic: A Cross-Sectional Study in Anhui Province, East China

**DOI:** 10.3389/fpsyg.2021.720911

**Published:** 2021-11-30

**Authors:** Wei-wei Chang, Liu Zhang, Li-ying Wen, Hong Su, Yue-long Jin

**Affiliations:** ^1^School of Public Health, Wannan Medical College, Wuhu, China; ^2^Department of Hospital Infection Management Office, Wuhu Hospital of Traditional Chinese Medicine, Wuhu, China; ^3^Department of Epidemiology and Health Statistics, School of Public Health, Anhui Medical University, Hefei, China

**Keywords:** college students, COVID-19, negative emotions, self-directed learning ability, online learning

## Abstract

**Background:** This study investigated the correlation between depression, anxiety, and stress among college students engaged in online learning during the coronavirus disease 2019 (COVID-19) pandemic and self-directed learning (SDL) ability, which could provide a scientific basis for mental health education of the college students.

**Methods:** A cross-sectional study was conducted among 5,558 students from two universities in Anhui province, East China. The Depression, Anxiety, and Stress Scale-21 (DASS-21) and the Self-directed Learning Ability Scale were used to conduct an online questionnaire survey.

**Results:** A total of 35.15, 36.32, and 17.24% of college students reported symptoms of depression, anxiety, and stress, respectively. Males and nonmedical students were at higher risks to suffer from depression, anxiety, and stress. In adjusted model, compared with Q1 of self-management ability, the odds ratio (OR) of the Q2, Q3, and Q4 were as follows: 0.635 (0.531–0.759), 0.504 (0.415–0.611), and 0.312 (0.248–0.392) for depression; 0.639 (0.535–0.764), 0.653 (0.540–0.789), and 0.421 (0.338–0.525) for anxiety; and 0.649 (0.523–0.805), 0.579 (0.457–0.733), and 0.482 (0.364–0.637) for stress. For information capability, decrease in risk was even more pronounced: Q2 (0.654, 0.540–0.794), Q3 (0.560, 0.454–0.690), and Q4 (0.233, 0.181–0.301) for depression; Q2 (0.781, 0.646–0.945), Q3 (0.616, 0.501–0.757), and Q4 (0.276, 0.216–0.353) for anxiety; and Q2 (0.444, 0.357–0.553), Q3 (0.454, 0.357–0.578), and Q4 (0.272, 0.202–0.368) for stress. Compared with the Q2 group of cooperation learning ability, cooperation learning ability quartiles were positively associated with depression (Q1: 1.382, 95% CI: 1.138–1.678), anxiety (Q4: 1.260, 95% CI: 1.008–1.576), and stress (Q1: 2.002, 95% CI: 1.583–2.532; Q3: 1.600, 95% CI: 1.252–2.044; Q4: 1.674, 95% CI: 1.243–2.255).

**Conclusion:** The prevalence of depression, anxiety, and stress among college students was high for those studying online at home during the COVID-19 pandemic, especially among nonmedical students and males. SDL ability was negatively associated with negative emotions of the college students during this period of online learning.

## Introduction

In December 2019, a new type of coronavirus pneumonia [coronavirus disease 2019 (COVID-19)] appeared and spread nationwide, attracting widespread attention from the international community (Hui et al., 2020). Facing the severe situation of epidemic prevention and control, a large number of university students who had returned to their hometowns remained there in strict self-isolation (Deng and Peng, 2020). In order to ensure public health safety during the growing epidemic turned pandemic and timely address the issue of students unable to engage in in-person learning, the Ministry of Education required universities to postpone the start of school in the spring of 2020 (Yu et al., 2021). Particularly, in a statement, it ordered “class suspension and no stop learning” (Sun and Su, 2020). This sudden change in learning style significantly impacted college students used to classroom learning (Yu et al., 2021). Moreover, lifestyle changes (e.g., extended vacations, online teaching, and restrictions on outside activities) affected their academic performance and aggravated negative emotional states such as anxiety and depression (Li W. et al., 2020; Zhao et al., 2020).

College students, in particular, are in a vulnerable transitional stage of shifting from relying on their parents to living independently. During this time, they typically begin to deal with all the kinds of social relationships and face various conflicts and pressures including the high level of academic pressure, employment stress, and the emotional problems (Liu et al., 2017; Zhang C. L. et al., 2020). As a result, Chinese college students are at high risk for some mental health problems. Evidence from a meta-analysis of 113 studies has shown that the overall prevalence of depression among Chinese university students was as high as 28.4% (Gao et al., 2020). Across different populations, the COVID-19, as a global public health event, has been shown to lead to psychological crises such as acute stress disorder, anxiety, and depression (Kang et al., 2020; Liem et al., 2020; Price et al., 2020). The COVID-19 pandemic has changed the daily life of Chinese college students. In order to prevent the spread of the COVID-19 pandemic, college students had to be quarantined at home, which may lead to the increase of social disconnection and the decrease of peer support (Deng and Peng, 2020), while lack of peer support is associated with depressive symptoms of the students (Sun et al., 2020). Several studies have shown that, among both the medical students and nonmedical students, the COVID-19 pandemic had a certain negative impact on their psychology during the period of home isolation (Tang et al., 2020a,b; Xie et al., 2020). Furthermore, college students could not return to school to study and the online teaching method replaced the traditional face-to-face teaching. One study from China indicated that learning burnout was highly prevalent in medical students who participated in online learning (Zhang et al., 2021). Over four-fifths (82.3%) of medical students who engaged in online learning suffered from moderate-to-high levels of stress (Wang et al., 2021). During the COVID-19 pandemic, as medical students and nonmedical students face the double pressure of a public health crisis and study, their emotional reactions might be more intense, thus increasing the risk of negative emotions such as anxiety and depression (Li L. et al., 2020; Wang Z. H. et al., 2020). Therefore, the mental health of medical and nonmedical students engaged in online learning should be evaluated.

Self-directed learning (SDL) refers to the initiative to judge learning needs, establish learning goals, select and implement appropriate learning strategies, and evaluate learning outcome with or without help from others (Levett-Jones, 2005). Online teaching formats with no in-class component require students to complete many learning tasks on their own under the arrangements of the school and the teacher. Thus, the ability to learn independently is especially important during the COVID-19 pandemic (Xu et al., 2020). SDL ability was an important determinant of academic performance (Tio et al., 2016), while mental health problems are clearly associated with lower academic functioning (Bruffaerts et al., 2018). Therefore, we propose the hypothesis that SDL ability of college students has a certain impact on their mental health.

Previous studies have mostly explored the influencing factors of psychological problems from learning pressure, interpersonal relationships, and the family environment (Shao et al., 2020; Tang et al., 2020c), but few studies have assessed such issues from the perspective of SDL ability of the students. Therefore, in this study, we aimed: (1) to investigate the negative emotions of college students engaged in online learning after the outbreak of the COVID-19 in China and (2) to explore the relationship between SDL ability and negative emotions among college students engaged in online learning. As such, this study can provide new findings for the research of mental health education of the college students.

## Materials and Methods

### Design and Subjects

This cross-sectional study was conducted in two colleges: Wannan Medical College (a medical school) and Anhui Engineering University (a nonmedical school) in Anhui province, China. There were 15,813 students enrolled in Wannan Medical College in the 2019/2020-2 semester including 25 majors with 4- or 5-year programs. There are 2–30 classes in each major and each class has 25–33 students. Students in years 4 and 5 were required to complete internships and, thus, did not participate in online learning. Therefore, the participants of this study were all the first-, second-, or third-year students. All the students in years 1–3 (10,923 students) were engaged in online learning. In this study, 170 classes (approximately 5,100 students) were randomly selected from 25 majors to complete the online survey. At the same time, about 1,500 students from 50 classes with some majors (the admission score of the college entrance examination is similar to that of Wannan Medical College) were randomly selected from Anhui University of Engineering University to complete the online survey.

During this period, students isolated themselves at home under the influence of the COVID-19 pandemic. A convenience sample was obtained via a web-based survey that was distributed through the Wenjuan platform, which is one of the most popular online survey platforms in China^1^ from June to July, 2020 (2019/2020-2 semester). The samples were obtained by the questionnaire spreading through network communication tools (such as WeChat and QQ group). All the students were aware of the purpose and significance of this survey and filled it out voluntarily. Ethical approval for this study was granted by the Ethics Committee of Wannan Medical College (LL-2020BH2086). A total of 5,558 valid questionnaires were collected in this survey including 2,585 males (2,585/5,558 = 46.51%).

### Survey Instrument

The self-designed online questionnaire included four parts: sociodemographic characteristics, online learning, the negative emotions, and SDL ability.

The basic information of college students included gender, age, grade, types of school, and birthplace. Online learning-related information included daily online learning time of the students, satisfaction of the students with teacher preparations, and satisfaction of the students with online learning effects.

The Depression, Anxiety, and Stress Scale-21 (DASS-21) was used to measure the negative emotions of college students experienced in the past week. A total of 21 items including three subscales: depression, anxiety, and stress. The DASS-21 was widely used in the screening of psychological conditions of the general population or clinical patients (Vignola and Tucci, 2014; Yohannes et al., 2019; Kuswanto et al., 2020; Fauzi et al., 2021). Each item used a four-point scoring from “0” (did not apply to me at all) to “3” (applied to me very much or most of the time). The final scores of each subscale are the sum of the scores of each subscale multiplied by 2. According to classification criteria, the negative emotions can be further divided into five levels (Lovibond and Lovibond, 1995): normal (0–9 points for depression, 0–7 points for anxiety, and 0–14 points for stress); mild (10–13 points for depression, 8–9 points for anxiety, and 15–18 points for stress); moderate (14–20 points for depression, 10–14 points for anxiety, and 19–25 points for stress); severe (21–27 points for depression, 15–19 points for anxiety, and 26–33 points for stress); and extremely severe (≥ 28 points for depression, ≥ 20 points for anxiety, and ≥ 34 points for stress). Depression, anxiety, and stress defined as score of ≥ 10, ≥ 8, and ≥ 15, respectively. The scale has good reliability and validity and can be used as an effective tool for investigating the mental health of Chinese college students (the Cronbach’s α was 0.81, 0.80, and 0.87 for the depression, anxiety, and stress subscale, respectively) (Xiong et al., 2021). In this study, the Cronbach’s α reliability coefficients for each subscale were also good (depression α = 0.900, anxiety α = 0.868, and stress α = 0.874) and the test–retest reliability was 0.971.

Self-directed learning ability of students was measured by using a validated Chinese version of the Self-directed Ability Scale of the college students. A total of 28 items included three subscales: (1) self-management ability, 10 items including the ability to determine learning needs, time management ability, and learning monitoring ability (e.g., “I often set learning goals”); (2) information capability, 11 items including information acquisition ability and information analysis and processing ability (e.g., “It is difficult for me to grasp the key points in my study”); and (3) cooperative learning ability, 7 items including the ability to communicate and ask for help (e.g., “When other student ask me for my learning experience, I can always say one or two points”). Each item was scored on a five-point Likert scale ranging from 1 (strongly disagree) to 5 (strongly agree). The total score of SDL ability was the sum of the score for each item. Higher scores indicate higher ability of SDL. The questionnaire had good reliability and validity (Lin and Jiang, 2004). In this study, the Cronbach’s α for self-management ability, information capability, and cooperative learning ability subscale was 0.802, 0.709, and 0.764, respectively, indicating a good internal consistency for each subscale.

### Data Analysis

The statistical analysis was performed by using the Statistical Package for the Social Sciences (SPSS) version 25 for Windows. The mean (SD) and percentage were used for descriptive analysis. First, the Kolmogorov–Smirnov tests were performed to assess the normality distribution of DASS-21 scores and SDL scores. Our results concluded that DASS-21 scores and SDL scores did not follow a normal distribution (*p* < 0.05). The Spearman’s rank correlation coefficients were used to evaluate the association between DASS-21 subscale scores and SDL subscale scores. The DASS-21 subscale scores were also divided into two groups of normal and abnormal (mild/moderate/severe/extremely severe) according to the DASS-21 User Manual (Lovibond and Lovibond, 1995). The univariate logistic regression analysis was used to explore the effects of sociodemographic variables and online learning variables on the negative emotions among college students.

Considering the conventional factors, which affect the negative emotions, we conducted a three-step multiple logistic regression modeling to analyze the association between SDL ability quartiles and negative emotions of the college students. Model 1 did not adjust the variables. Model 2 adjusted for gender, age, school type, and grade. Model 3 adjusted for gender, age, school type, grade, birthplace, daily online learning time of the students, satisfaction of the students with teacher preparations, and satisfaction of the students with online learning effects. In the three multiple logistic regression models, the scores of self-management ability, information capability, and cooperation learning ability were categorized into four levels (Q1, Q2, Q3, and Q4) by using the quartiles (P_25_, P_50_, and P_75_) as cutoff values. The self-management ability and information capability took Q1 as the reference, while the cooperative ability took Q2 as the reference for the multivariate regression analysis. Model results are presented as odds ratios (ORs) with 95% CIs, R-squared (R^2^), and adjusted R-squared (AR^2^) (Cohen, 1988).

## Results

### Sample Characteristics

A total of 5,558 college students were enrolled in this study (mean age: 20–30 years) including 4,115 medical students and 1,443 nonmedical students. In terms of grade, first-, second-, and third-year students accounted for 32.58, 37.08, and 30.34%, respectively (Table 1).

**TABLE 1:** Association among sociodemographic variables and mental health impact (*n* = 5,558).

**Variables**	***n* (%)**	**Depression**				**Anxiety**				**Stress**			
		***R* ^2^**	**AR^2^**	***OR***	**95% CI**	***R* ^2^**	**AR^2^**	***OR***	**95% CI**	***R* ^2^**	**AR^2^**	***OR***	**95% CI**
**Gender**													
Male	2,585 (46.51)	0.011	0.015	1.551^**^	1.388-1.732	0.013	0.018	1.614^**^	1.446-1.802	0.003	0.004	1.310^**^	1.140-1.506
Female	2,973 (53.49)			Ref.				Ref.				Ref.	
**Types of school**													
Non-medical	1,443 (25.96)	0.013	0.018	1.712^**^	1.514-1.936	0.014	0.020	1.757^**^	1.554-1.985	0.013	0.022	1.933^**^	1.668-2.240
Medical	4,115 (74.04)			Ref.				Ref.				Ref.	
**Grade**													
Third-year	1,686 (30.34)	0.001	0.002	1.192^*^	1.037-1.370	0.0002	0.0003	1.033	0.905-1.178	0.005	0.008	1.406^**^	1.180-1.675
Second-year	2,061 (37.08)			1.117	0.978-1.276			1.081	0.942-1.241			1.601^**^	1.338-1.916
First-year	1,811 (32.58)			Ref.				Ref.				Ref.	
**Birthplace**													
City	1,234 (22.20)	0.001	0.001	0.893	0.776-1.027	0.001	0.002	0.826^*^	0.718-0.950	0.001	0.001	1.040	0.875-1.237
Town	1,389 (24.99)			0.880	0.769-1.007			0.909	0.796-1.038			0.855	0.718-1.017
Village 2935 (52.81)	2,935 (52.81)			Ref.				Ref.				Ref.	

*P-value < 0.05. **P-value < 0.001. R^2^, R-squared; AR^2^, Adjusted R-squared; CI, Confidence interval.

Nearly half of participants studied online for 4–6 h per day (47.64%) and 520 (9.36%) students studied online for less than 2 h per day. A total of 785 students (14.12%) were not satisfied with online teaching. The vast majority of students (71.81%) were satisfied with the preparation of teachers for online teaching. Other basic information of 5,558 students was listed in Tables 1, 2.

**TABLE 2:** Association among online learning variables and mental health impact (*n* = 5,558).

**Variables**	***n* (%)**		**Depression**				**Anxiety**				**stress**			
		***n* (%)**	***R* ^2^**	**AR^2^**	***OR***	**95% CI**	***R* ^2^**	**AR^2^**	***OR***	**95% CI**	***R* ^2^**	**AR^2^**	***OR***	**95% CI**
Satisfaction with teacher preparations														
	Dissatisfied	115 (2.07)	0.037	0.052	4.181^**^	2.843-6.148	0.034	0.046	3.626^**^	2.475-5.313	0.016	0.027	4.796^**^	3.288-6.996
	General	1452 (26.12)			2.295^**^	2.029-2.597			2.204^**^	1.950-2.493			1.643^**^	1.411-1.912
	Satisfied	3991 (71.81)			Ref.				Ref.				Ref.	
Online learning time (hour)														
	0–2	520 (9.36)	0.017	0.023	2.418^**^	1.961-2.982	0.013	0.018	1.984^**^	1.611-2.443	0.008	0.013	2.094^**^	1.632-2.686
	2-	1126 (20.26)			1.537^**^	1.298-1.820			1.498^**^	1.268-1.770			1.423^**^	1.149-1.763
	4-	2648 (47.64)			1.085	0.939-1.255			1.004	0.871-1.158			1.068	0.885-1.288
	≥ 6	1264 (22.74)			Ref.				Ref.				Ref.	
Satisfaction with online learning effects														
	Dissatisfied	785 (14.12)	0.021	0.029	2.354^**^	1.986-2.790	0.015	0.021	2.081^**^	1.758-2.462	0.022	0.036	3.093^**^	2.537-3.771
	General	2656 (47.79)			1.667^**^	1.473-1.886			1.502^**^	1.330-1.696			1.396^**^	1.185-1.644
	Satisfied	2117 (38.09)			Ref.				Ref.				Ref.	

**P-value < 0.001. R^2^, R-squared; AR^2^, Adjusted R-squared; CI, Confidence interval.

### Prevalence of Depression, Anxiety, and Stress Among College Students

According to the DASS-21 subscale grading standards, the prevalence of depression (score ≥ 10), anxiety (score ≥ 8), and stress (score ≥ 15) were 35.15, 36.32, and 17.24%, respectively (Table 3). Among the students in a state of depression, 635 (11.42%) students suffered from mild depression and 1,005 (18.08%) students suffered from moderate depression. Among the students in a state of anxiety, 377 (6.78%) students suffered from mild anxiety and 1,147 (20.64%) students suffered from moderate anxiety. For the stress subscale, 502 (9.03%) students were considered to suffer from mild stress and 275 (4.95%) students were considered to suffer from moderate stress. The proportions of students with different levels of the DASS-21 subscales were shown in Table 3.

**TABLE 3:** Distribution of all subjects’ grades in each DASS-21 subscale [*n*(%)] (*n* = 5,558).

**Group**	**Depression**	**Anxiety**	**Stress**
No symptoms	3,604(64.85)	3,539(63.68)	4,600(82.76)
Mild	635 (11.42)	377 (6.78)	502 (9.03)
Moderate	1,005(18.08)	1,147(20.64)	275 (4.95)
Severe	189 (3.40)	237 (4.26)	126 (2.27)
Extremely severe	125 (2.25)	258 (4.64)	55 (0.99)

### Sociodemographic Variables, Online Learning Variables, Self-Directed Learning Ability, and Mental Health Impact

Males were at higher risks than females to suffer from depression (OR = 1.551, 95% CI = 1.388–1.732), anxiety (OR = 1.614, 95% CI = 1.446–1.802), and stress (OR = 1.310, 95% CI = 1.140–1.506) with low effect size. Nonmedical students were at higher risks than medical students to suffer from depression (OR = 1.712, 95% CI = 1.514–1.936), anxiety (OR = 1.757, 95% CI = 1.554–1.985), and stress (OR = 1.933, 95% CI = 1.668–2.240) with low effect size. Third-year students were positively associated with the risks of depression (OR = 1.192, 95% CI = 1.037–1.370) and stress (OR = 1.406, 95% CI = 1.180–1.675) (Table 1).

Dissatisfaction with online learning effects was associated with the risks of depression (OR = 2.354, 95% CI: 1.986–2.790), anxiety (OR = 2.081, 95% CI = 1.758–2.462), and stress (OR = 3.093, 95% CI = 2.537–3.771) with low effect size. Statistically significant difference was also found for other online learning variables under study (Table 2).

All the subscale scores of SDL ability were negatively related with depression, anxiety, and stress (*p* < 0.001, Table 4), with medium or low effective sizes.

**TABLE 4:** Spearman’s correlation coefficients between DASS-21 subscale scores and SDL subscale scores among students.

	**Depression**	**Anxiety**	**Stress**
Self-management ability	−0.397^**^	−0.306^**^	−0.297^**^
Information capability	−0.420^**^	−0.341^**^	−0.331^**^
Cooperative learning ability	−0.345^**^	−0.247^**^	−0.254^**^

**P-value < 0.001.

### Associations of Self-Directed Learning Ability With Depression, Anxiety, and Stress (Multivariate Analysis)

Taking depression, anxiety, and stress symptoms of college students as dependent variables (0 = No, 1 = Yes), the logistic regression analysis was conducted to explore the association of SDL ability with the negative emotions of college students.

For both the self-management ability and information capability, compared to students with the lowest quartile (Q1), increasing scores conferred a stepwise decrease in risk for depression, anxiety, and stress. The association remained significant in adjusted models (*p* < 0.01, with medium or low effective sizes). For self-management ability in model 3, the risk was as follows (OR, 95% CI): Q2 (0.635, 0.531–0.759), Q3 (0.504, 0.415–0.611), and Q4 (0.312, 0.248–0.392) for depression; Q2 (0.639, 0.535–0.764), Q3 (0.653, 0.540–0.789), and Q4 (0.421, 0.338–0.525) for anxiety; and Q2 (0.649, 0.523–0.805), Q3 (0.579, 0.457–0.733), and Q4 (0.482, 0.364–0.637) for stress. For information capability in model 3, decrease in risk was even more pronounced: Q2 (0.654, 0.540–0.794), Q3 (0.560, 0.454–0.690), and Q4 (0.233, 0.181–0.301) for depression; Q2 (0.781, 0.646–0.945), Q3 (0.616, 0.501–0.757), and Q4 (0.276, 0.216–0.353) for anxiety; and Q2 (0.444, 0.357–0.553), Q3 (0.454, 0.357–0.578), and Q4 (0.272, 0.202–0.368) for stress (Table 5).

**TABLE 5:** The results of logistic regression analysis of SDL ability and depression, anxiety, and stress among students (*n* = 5,558).

**Dependent variable**	**Independent variable**	**Group**	**Prevalence (%)**	**Model 1**				**Model 2**				**Model 3**			
				***R*^2^/AR^2^**	***P***	***OR***	**95% CI**	**R^2^/A*R*^2^**	***P***	***OR***	**95% CI**	**R^2^/AR^2^**	***P***	***OR***	**95% CI**
Depression	Self- management ability	Q1	783 (58.78)	0.140/0.192		Ref.		0.159/0.219		Ref.		0.174/0.240		Ref.	
		Q2	454 (41.58)		<0.001	0.595	0.500-0.708		<0.001	0.595	0.498-0.710		<0.001	0.635	0.531-0.759
		Q3	436 (30.47)		<0.001	0.459	0.381-0.554		<0.001	0.465	0.384-0.562		<0.001	0.504	0.415-0.611
		Q4	281 (16.50)		<0.001	0.290	0.233-0.363		<0.001	0.291	0.233-0.365		<0.001	0.312	0.248-0.392
	Information capability	Q1	497 (59.74)			Ref.				Ref.				Ref.	
		Q2	736 (46.06)		<0.001	0.682	0.565-0.822		<0.001	0.676	0.559-0.817		<0.001	0.654	0.540-0.794
		Q3	491 (32.82)		<0.001	0.551	0.450-0.675		<0.001	0.549	0.446-0.675		<0.001	0.560	0.454-0.690
		Q4	230 (14.09)		<0.001	0.233	0.182-0.299		<0.001	0.220	0.171-0.284		<0.001	0.233	0.181-0.301
	Cooperative learning ability	Q2	569 (45.56)			Ref.				Ref.				Ref.	
		Q1	554 (55.57)		0.001	1.383	1.147-1.668		0.003	1.331	1.100-1.611		0.001	1.382	1.138-1.678
		Q3	482 (28.59)		0.616	0.954	0.794-1.147		0.516	0.94	0.779-1.133		0.822	0.978	0.809-1.183
		Q4	349 (21.46)		0.076	1.224	0.979-1.531		0.196	1.162	0.926-1.459		0.051	1.257	0.999-1.583
Anxiety	Self-management ability	Q1	744 (55.86)	0.098/0.134		Ref.		0.120/0.165		Ref.		0.135/0.184		Ref.	
		Q2	432 (39.57)		<0.001	0.602	0.507-0.715		<0.001	0.601	0.504-0.716		<0.001	0.639	0.535-0.764
		Q3	495 (34.59)		<0.001	0.596	0.496-0.716		<0.001	0.6	0.498-0.723		<0.001	0.653	0.540-0.789
		Q4	348 (20.43)		<0.001	0.390	0.315-0.483		<0.001	0.39	0.313-0.485		<0.001	0.421	0.338-0.525
	Information capability	Q1	455 (54.69)												
		Q2	756 (47.31)		0.021	0.805	0.670-0.968		0.020	0.800	0.663-0.965		0.011	0.781	0.646-0.945
		Q3	523 (34.96)		<0.001	0.599	0.490-0.731		<0.001	0.602	0.491-0.738		<0.001	0.616	0.501-0.757
		Q4	285 (17.46)		<0.001	0.272	0.214-0.346		<0.001	0.261	0.204-0.333		<0.001	0.276	0.216-0.353
	Cooperative learning ability	Q2	580 (46.44)			Ref.				Ref.				Ref.	
		Q1	498 (49.95)		0.295	1.104	0.917-1.328		0.534	1.062	0.879-1.282		0.332	1.100	0.908-1.332
		Q3	532 (31.55)		0.824	0.980	0.818-1.174		0.728	0.968	0.805-1.163		0.931	1.008	0.837-1.214
		Q4	409 (25.15)		0.058	1.233	0.993-1.532		0.164	1.170	0.938-1.459		0.042	1.260	1.008-1.576
Stress	Self-management ability	Q1	380 (28.53)	0.068/0.113		Ref.		0.085/0.142		Ref.		0.098/0.163		Ref.	
		Q2	208 (19.05)		<0.001	0.631	0.513-0.778		<0.001	0.609	0.493-0.754		<0.001	0.649	0.523-0.805
		Q3	203 (14.19)		<0.001	0.522	0.414-0.657		<0.001	0.532	0.421-0.672		<0.001	0.579	0.457-0.733
		Q4	167 (9.81)		<0.001	0.435	0.331-0.572		<0.001	0.445	0.338-0.587		<0.001	0.482	0.364-0.637
	Information capability	Q1	316 (37.98)			Ref.				Ref.				Ref.	
		Q2	272 (17.02)		<0.001	0.456	0.369-0.563		<0.001	0.448	0.361-0.555		<0.001	0.444	0.357-0.553
		Q3	229 (15.31)		<0.001	0.444	0.352-0.561		<0.001	0.442	0.349-0.562		<0.001	0.454	0.357-0.578
		Q4	141 (8.64)		<0.001	0.267	0.199-0.358		<0.001	0.254	0.188-0.342		<0.001	0.272	0.202-0.368
	Cooperative learning ability	Q2	190 (15.21)			Ref.				Ref.				Ref.	
		Q1	327 (32.80)		<0.001	2.099	1.674-2.630		<0.001	2.006	1.594-2.526		<0.001	2.002	1.583-2.532
		Q3	260 (15.42)		0.001	1.612	1.270-2.047		<0.001	1.554	1.219-1.981		<0.001	1.600	1.252-2.044
		Q4	181 (11.13)		<0.001	1.665	1.245-2.227		0.004	1.535	1.143-2.063		0.001	1.674	1.243-2.255

*R^2^, R-squared; AR^2^, Adjusted R-squared; CI, Confidence interval. Model 1: Crude; Model 2: adjust for gender, age, school type and grade; Model 3: adjust for gender, age, school type, grade, birthplace, students’ daily online learning time, students’ satisfaction with teacher preparations, and students’ satisfaction with online learning effects.*

In model 3, compared with the Q2 (P_25_ to P_50_) group of cooperation learning ability, cooperation learning ability quartiles were positively associated with depression (Q1: 1.382, 95% CI: 1.138–1.678), anxiety (Q4: 1.260, 95% CI: 1.008–1.576), and stress (Q1: 2.002, 95% CI: 1.583–2.532; Q3: 1.600, 95% CI: 1.252–2.044; Q4: 1.674, 95% CI: 1.243–2.255) (Table 5).

## Discussion

For college students accustomed to face-to-face teaching, the completely online teaching method might aggravate their psychological problems. This was the first study to investigate depression, anxiety, and stress among college students engaged in online learning after the outbreak of the COVID-19 in China. During online remote learning, 35.15, 36.32, and 17.24% of college students reported symptoms of depression, anxiety, and stress. Depression (18.08%) and anxiety (20.64%) were both moderately predominant, while stress was predominantly mild (9.03%). Meanwhile, an online questionnaire survey in Bangladesh by using the DASS-21 survey showed that 46.92% of students had depression, 33.3% of students had anxiety, and 28.5% of students had stress, which significantly differ from this study (Khan et al., 2020). As we all know, the severity of the COVID-19 pandemic varies in different countries, which may explain differences in mental health of the people. Our finding also differs from that of another previous study (Cao et al., 2020) that showed that 24.9% of medical students were experiencing anxiety (21.3% mild anxiety) during the COVID-19 pandemic. In this study, the Generalized Anxiety Disorder-7 (GAD-7) Scale was used to measure the mental health of college students and the target population was those attending medical college. A large cross-sectional online survey from January 31, 2020 to February 5, 2020 among college students in China showed that the prevalence of anxiety and depression symptom was 7.7 and 12.2%, respectively (Wang C. et al., 2020). The prevalence of symptoms of depression, anxiety, and stress is not consistent in different studies. This may be due to different measurement tools, different research participants, and different school types (Luo et al., 2021). Beside, mental health state of the people appears to change at different stages of the COVID-19 pandemic. A longitudinal survey in China reported that acute stress, anxiety, and depressive symptoms among college students showed a significant increase in the remission period of the COVID-19 compared with the initial outbreak (Li et al., 2021). The above research results indicate that college students experience mental health problem due to the COVID-19 outbreak. Thus, it is necessary for colleges and universities to provide some mental health education and guidance. It is noteworthy that 5.65% of students reported severe to extremely severe depression and 8.90% of students reported severe to extremely severe anxiety. This might be due to the fact that online learning was nearing completion at the time of survey. Students often face greater learning pressure at the end of the semester. Severe depression, anxiety, and stress have been identified as the main independent risk factors for suicidal behavior in college students (Guo et al., 2019). Therefore, it is necessary to pay special attention to this particular student cohort.

In this study, the prevalence of depression and anxiety in males was higher than that of females, which differed from the results of other studies conducted during the COVID-19 pandemic (Gao et al., 2020; Luo et al., 2021). This may be because social expectations of men are higher in China. Self-regulation of learning is considered as one of the key capabilities during online learning (Alt and Naamati-Schneider, 2021), while the SDL ability of males was lower than that of females (Xu and Li, 2019). Therefore, males experienced a higher psychological impact of the COVID-19 than females engaged in online learning. We found that during the online learning period of the COVID-19 pandemic, medical students had better mental health than nonmedical students. This finding is consistent with a study conducted on colleges in China suggesting that medical students experienced a lower psychological impact of the COVID-19 than nonmedical students (Xie et al., 2020). This might be due to the fact that medical students had richer medical background knowledge and a better understanding of the elements of disease. The curriculum system of medical students included courses such as health education and mental hygiene, which could better prepare and teach them how to scientifically respond to public health emergencies (Xie et al., 2020). Several studies showed that the students increased their knowledge and felt that they were better prepared for the epidemic after health education intervention (Siddle et al., 2016; Patel et al., 2018). The result in the study reported by Wang et al. demonstrated that well-established online learning environment support was critical for relieving the negative impacts of the COVID-19 on the psychosocial health among medical students (Wang et al., 2021). Thus, the school should start the COVID-19 pandemic situation online mental health service platform and provide self-protection methods about the COVID-19 pandemic, which is important to counteract feelings of anxiety and depression.

Consistent with our hypothesis, for self-management ability and information capability, compared with the Q1 group, increasing scores conferred a stepwise decrease in risk for depression, anxiety, and stress. Learning management ability is an important component of learning ability and a necessary part of academic success of the students (Zhang Q. et al., 2020). A longitudinal study indicated that academic achievement was negative predictors of depression (Liu et al., 2018). Students with low scores of self-management ability cannot effectively use time and goal setting to cultivate their comprehensive quality and they are prone to psychological activities with poor self-efficacy when learning online (Chiu et al., 2016; Wu et al., 2020). As the core component of individual self-regulation system, self-efficacy plays an important role in mental health of the students (Schönfeld et al., 2016; Grøtan et al., 2019). Therefore, during the period of online learning, we can improve learning efficiency of the students by paying attention to their learning management, so as to reduce the occurrence of negative emotions of the students. Students with poor information capability are prone to confusion when facing multichannel information-based teaching. Recent literature has pointed that 32.9% of Chinese college students were unfamiliar operation of the learning platform and unfamiliar learning platform operation of the students was a risk factor for psychological stress (Yu et al., 2021). Another large survey in China demonstrated that students were most familiar with recorded broadcast courses (36.84%) and massive open online courses (MOOCs) (35.00%) and they considered the support and service of platforms to be insufficient (Wang C. et al., 2020). Therefore, in order to effectively improve online medical education, teachers should choose the teaching platform familiar to students and use the information platform to carry out curriculum design. At the same time, it is necessary for schools to provide more support and training for teaching strategies of the teachers and information skills of the students.

Multifactor analysis of this study found that the high or low scores of learning cooperation ability might both be the high-risk factors for negative emotions. In the process of learning, learning from each other can promote the progress of teaching (Zhang et al., 2019). Most students cannot adapt to the fully online teaching model in a short time (Yu et al., 2021). Prevention and isolation measures implemented during the COVID-19 pandemic have drastically changed the learning environment and cooperation methods. Students with low score in cooperative learning ability cannot share the online learning experience with other students, which may lead to the increase of social disconnection and the decrease of peer support (Deng and Peng, 2020). A cluster randomized trial found that cooperative learning mainly affected cognitive empathy through the improvement of peer relationship (Van Ryzin and Roseth, 2019). Perceived available peer support negatively contributed to depressive symptoms (Sun et al., 2020). Students with high scores in cooperative learning ability usually adopt face-to-face cooperative learning methods such as collective self-study, on-site discussions, and communication (Thibaut and Schroeder, 2020). The experience and methods of cooperative learning in ordinary times cannot be realized online, which reduces the efficiency of learning and leads to negative emotions. Interpersonal relationship is the most important component of cooperative learning (Dale et al., 2020). Cooperation is a quality that requires teachers to cultivate patiently and train for a long time. Teachers should improve interpersonal relationship and social skills of the students in classroom learning and after school life.

## Limitations

There are limitations of this study, which should be considered when interpreting these findings. First, this study was cross-sectional and it cannot draw causal conclusion. In addition, potential selection bias from the sampling technique and reporting bias in the self-administered survey may affect the results. Second, the way students fill in the questionnaire is through the internet, which reduces the recovery rate and efficiency of the questionnaire. Additionally, other confounding factors (such as family income, physical exercise, and smoking) were not collected in this study.

## Conclusion

The prevalence of depression, anxiety, and stress among college students was high for those studying online at home during the COVID-19 pandemic, especially among nonmedical students and males. SDL ability was negatively associated with negative emotions of the college students during this period of online learning. In addition to offering mental health education courses in schools, teachers should pay attention to cultivating SDL ability of the students throughout the teaching process. At the same time, schools should also take measures to enable parents of the students to realize the importance of SDL ability for students and to consider how this change in learning format may negatively impact mental health of the students.

## Data Availability Statement

The raw data supporting the conclusions of this article will be made available by the authors, without undue reservation.

## Ethics Statement

The study was approved by School of Public Health of Wannan Medical College. The participants provided their written informed consent to participate in this study. Written informed consent was obtained from the individual(s), and minor(s)’ legal guardian/next of kin, for the publication of any potentially identifiable images or data included in this article.

## Author Contributions

W-WC, HS, and Y-LJ contributed to the overall design, article selection, review, and manuscript preparation. LZ and L-YW contributed to investigation. W-WC contributed to writing original manuscript and submission. All authors contributed to the article and approved the submitted version.

## Conflict of Interest

The authors declare that the research was conducted in the absence of any commercial or financial relationships that could be construed as a potential conflict of interest.

## Publisher’s Note

All claims expressed in this article are solely those of the authors and do not necessarily represent those of their affiliated organizations, or those of the publisher, the editors and the reviewers. Any product that may be evaluated in this article, or claim that may be made by its manufacturer, is not guaranteed or endorsed by the publisher.

## Footnotes

1
https://www.wenjuan.com/list/
